# Functional connectivity density alterations in middle‐age retinal detachment patients

**DOI:** 10.1002/brb3.1783

**Published:** 2021-03-01

**Authors:** Yi Shao, Lin Yang, Pei‐Wen Zhu, Ting Su, Xue‐Zhi Zhou, Biao Li, Wen‐Qing Shi, Qi Lin, You‐Lan Min, Qing Yuan, Lei Ye, Qiong Zhou

**Affiliations:** ^1^ Department of Ophthalmology The First Affiliated Hospital of Nanchang University Nanchang China; ^2^ Eye Institute of Xiamen University Fujian Provincial Key Laboratory of Ophthalmology and Visual Science Xiamen, Fujian Province China; ^3^ Department of Ophthalmology Xiangya Hospital Central South University Hunan Province Changsha China

**Keywords:** functional connectivity density, magnetic resonance imaging, Middle‐age, retinal detachment

## Abstract

**Objective:**

Middle‐age patients with retinal detachment (RD) exhibit a loss of visual information, and previous studies of functional magnetic resonance imaging (fMRI) have demonstrated abnormal spontaneous activity in the RD brain. Therefore, this study assessed changes in local functional connectivity density (lFCD) and long‐range functional connectivity density (longFCD) in middle‐age RD patients during resting‐state FC.

**Methods:**

In total, 32 middle‐age patients with RD (18 men and 14 women), and 32 age‐, sex‐, and education‐matched normal controls (NCs) (18 men and 14 women) were recruited and underwent functional magnetic resonance examination in the resting state. Two‐sample *t* test was performed to compare lFCD and longFCD between groups. Receiver operating characteristic (ROC) curves were generated to distinguish middle‐age RD patients from NCs.

**Results:**

Compared with NCs, middle‐age RD patients demonstrated increased lFCD values in the right inferior temporal gyrus, and increased longFCD values in the bilateral inferior frontal gyri, left superior and middle frontal gyrus, bilateral inferior temporal gyri, and left cerebellum posterior lobe. Middle‐age RD patient exhibited decreased lFCD values in the left cuneus, right lingual gyrus, and left middle frontal gyrus. They also had lower longFCD values in the left lingual gyrus and left inferior occipital gyrus. ROC curve analysis showed excellent accuracy of the specific areas under the curve.

**Conclusion:**

Our results reveal that middle‐age RD patients exhibited variations of binarized lFCD and longFCD in specific brain areas, which provides insight into the pathological mechanism of RD patients with acute visual loss.

## Graphical abstract

We demonstrated that the RD patients showed variations of binarized IFCD and longFCD in vision and vision‐related brain regions, which can consummate the pathological mechanism of RD patients with acute visual information loss.

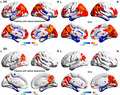

## 1 INTRODUCTION

**FIGURE 1:**
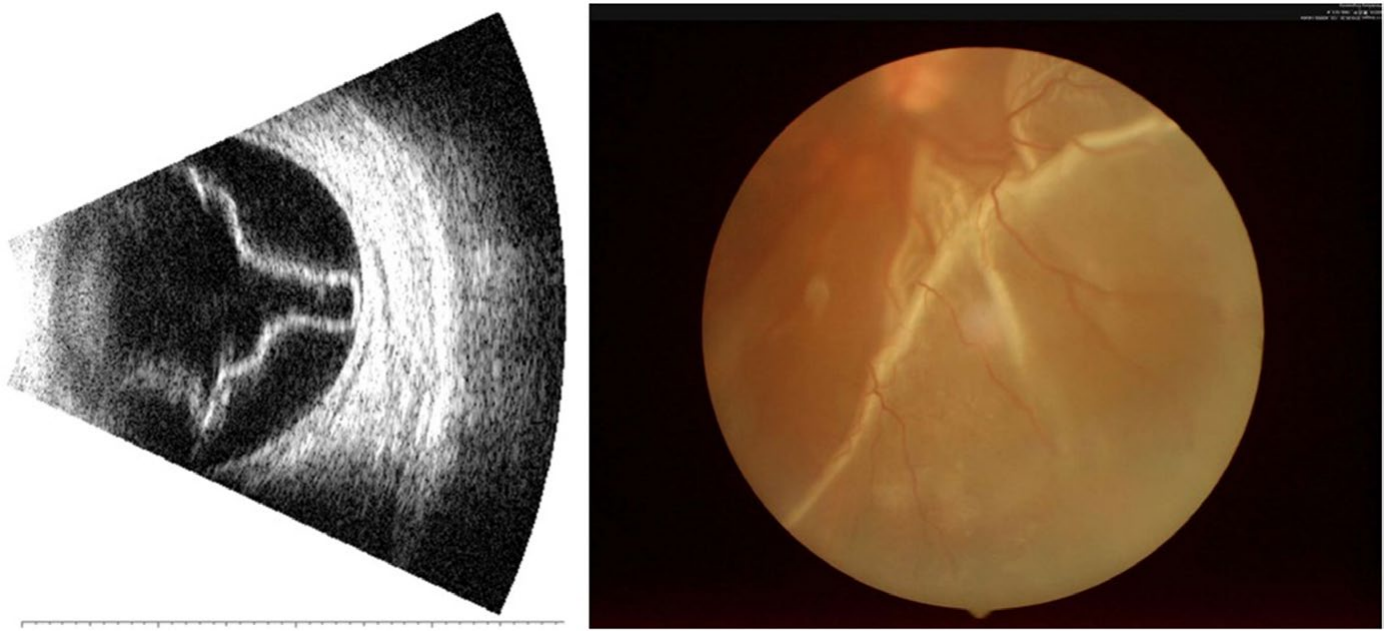
Example of RD seen on B‐ultrasound (left) and fundus (right)

Retinal detachment (RD) (Figure 1) refers to separation between the neural retina and retinal pigment epithelium; this leads to vision loss and increases the physical distance between photoreceptor cells and the retinal pigment epithelium and choriocapillaris (Fisher, et al., 2010). The main risk factors for middle‐age RD are uveitis (Amer, Nalci, & Yalcindag, 2017), high myopia (Baba et al., 2003), vitreous detachment (Fincham et al., 2018), and ocular trauma (Hoogewoud et al., 2016). Foveal detachment can rapidly damage central visual acuity (VA), and surgery is the main treatment (García‐Arumí et al., 2013). RD is typically diagnosed with B‐ultrasound and optical coherence tomography (OCT). Compared with B‐ultrasound, OCT is superior for observing the microstructural retina changes. The regional homogeneity method has been used to observe changes in brain neural homogeneity in middle‐age RD patients to understand their relationships with clinical features (Huang et al., 2017). Resting‐state functional connectivity (rs‐FC) analysis is a useful method to evaluate spontaneous functional organization though FC, and it gauges the temporal correlation between the time series of the blood oxygen level‐dependent (BOLD) signals of two brain regions (Tomasi et al., 2010). rs‐FC has been successfully applied to study depression (Klumpp, Hosseini, & Phan, 2018; Maglanoc et al., 2019), Alzheimer's disease (Hata et al., 2016), Parkinson's disease (Hu et al., 2017), and primary open‐angle glaucoma (Dai et al., 2013).

Recently, we have observed close correlation between the default mode network functional connections and visual function impairments of RD patients (Su et al., 2018). However, alterations in the local FC density (lFCD) and long‐range FC density (longFCD) have not been revealed. To investigate the effect of middle‐age RD patients on visual pathway and cortex, we applied rs‐FC to assess differences in local FC density (lFCD) and long‐range FC density (longFCD). We hypothesized that the lFCD and longFCD would be altered in middle‐age RD patients and that the spatial distribution of abnormal lFCD or longFCD and the abnormal pattern (increased or decreased) of lFCD or longFCD would be different in middle‐age RD patients compared with normal controls. Identifying unique changes in middle‐age RD patients could clarify the pathological mechanism and guide new treatment strategies.

## 2 PATIENTS AND METHODS

### 2.1 Subjects

We recruited 32 middle‐age RD patients (18 men and 14 women) who were treated in the First Affiliated Hospital of Nanchang University and examined by a single experienced ophthalmologist. The inclusion criteria were as follows: (1) middle‐age RD occurred within 1 month, (2) macular detachment, and (3) no comorbid ocular diseases (e.g., strabismus or amblyopia).The exclusion criteria were as follows: (1) systemic disease (e.g., cardiovascular disease or cerebral disease) and (2) psychiatric disorders (e.g., delusional or depressive disorder).

We also enrolled 32 age‐, sex‐, and education‐matched normal controls (NCs; 18 men, 14 women). The inclusion criteria were as follows: 1) normal brain parenchyma on cranial magnetic resonance imaging (MRI); 2) VA ≥ 1.0 and without any other ocular disease (e.g., retinitis pigmentosa, strabismus); and 3) no psychological issues (e.g., delusional disorder).

All research methods were approved by the committee of the medical ethics of the First Affiliated Hospital of Nanchang University, and participants were explained the intent, method, and potential risks, and they signed an informed consent form.

### 2.2 MRI data acquisition and data analysis

MRI was performed on a 3.0 Tesla (T) system (Magnetom TRIO, Siemens Medical; Erlangen, Germany). High‐resolution T1‐ and T2‐weighted imaging sequences were implemented with the following parameters: TR = 1,900 ms, TE = 2.26 ms, thickness = 1.0 mm, gap = 0.5 mm, FOV = 250 mm × 250 mm, matrix = 256 × 256, flip angle = 9°, 176 sagittal slices.

In the resting‐state scan session, a gradient echo‐planar imaging sequence was used to collect 240 functional volumes utilizing the parameters below: repetition time (TR), 2000 ms; echo time (TE), 30ms; slice gap, 1mm; flip angle (FA), 90. Matrix size 64*64; 35 oblique slices parallel to the AC‐PC line were acquired with interleaved acquisition. High‐resolution T1‐weighted anatomical images were acquired using a sagittal FSPGR‐BRAVO sequence with the following parameters: TR,8.208 s; inversion time (T1), 450 ms; TE, 3.22 ms; FA, 12; FOV, 240*240 mm; voxel size, 0.5 mm*0.5 mm*1 mm.

To balance the MRI scanning signal, the first 10 image was deleted. Based on MATLAB 2010a (MathWorks, Natick, MA, USA), the rest of the data preprocessing was performed by Data Processing & Analysis for Brain Imaging (DPABI 2.1, http://rfmri.org/DPABI) toolbox, including slice timing, head‐motion correction, and spatial normalization. Participants who had more than 1.5 mm maximum translation in x, y, or z directions and 1.5° degree of motion rotation were excluded from further analyses. The Friston 24 head‐motion parameter model was used to regress out head‐motion effects, based on recent work showing that higher‐order models benefit from their removal (Satterthwaite et al., 2013; Yan et al., 2013). Linear regression was performed to remove other sources of spurious covariates and their temporal derivatives, including the global mean signal and the white matter and cerebrospinal fluid signals. Functional MRI images were normalized spatially to the Montreal Neurological Institute (MNI) space and resampled at a resolution of 3 × 3×3 mm^3^ after head‐motion correction. Then, the time series for each voxel was temporally bandpass‐filtered (0.01–0.1 Hz) and linearly detrended to reduce low‐frequency drift, and physiological high‐frequency breathing and heart noise.

### 2.3 Calculation of lFCD and longFCD Maps

FCD maps contained long‐ and short‐range data for each individual, the short‐range FCD equates with local FCD, and all of them were calculated in a gray matter mask. Based on the Pearson correlations between the time course of a given voxel and that of other voxels, the number of functional connections of a given voxel was considered as a degree of a node in a binary graph. The detailed procedure for computing lFCD and longFCD is published elsewhere (Faivre et al., 2016). First, we defined FC in the whole brain between a given voxel with every other voxel, with a correlation threshold of *r* > 0.25 (Hata et al., 2016). We employed the threshold of *r* = 0.3 to calculate the FCD maps. Second, the lFCD and longFCD were defined on the basis of the neighborhood strategy, which means that voxels that met the correlation threshold of *r* = 0.30 inside and outside their neighborhood (radius sphere ≤6 mm and >6 mm) were defined as lFCD and longFCD, respectively. To improve normality, we divided the lFCD and longFCD maps by the mean value for the whole brain of each subject and converted the values to Z scores. Finally, SPM8 software was used to generate the lFCD and longFCD maps, which were spatially smoothed with a Gaussian kernel of 6 × 6×6 mm 3 full width at half maximum.

### 2.4 Ophthalmic testing

The international standard logarithmic VA chart was utilized for visual test analysis. Middle‐age RD patients were diagnosed by B‐ultrasound and spectral domain OCT.

### 2.5 Statistical analysis

Independent‐sample *t* tests were performed using SPSS version 16.0 (SPSS Inc, Chicago, IL, USA) to analyze the cumulative clinical measurements, including RD duration. Differences were considered significant at *p* < .05.

One‐sample *t* test was performed to extract the lFCD and longFCD value results across the subjects within each group and to examine the significant intragroup distribution. Differences were considered significant at *p* < .05. Statistical analysis of general linear model was carried out using the SPM8 toolkit. The two‐sample *t* tests were used to examine the differences of the FCD maps between the middle‐age RD groups and the NCs (two‐tailed, voxel‐level *p* < .01, Gaussian random field theory (GRF) correction, cluster‐level *p* < .05).

Receiver operating characteristic (ROC) curves were generated to distinguish the average lFCD from longFCD values in the various brain regions between the two groups. Differences were considered significant at *p* < .05.

## 3 RESULTS

### 3.1 Demographics and visual measurements

There were no apparent differences in weight (*p* = .87) or age (*p* = .89), but we observed significant differences in best‐corrected VA‐right (*p* = .001) and best‐corrected VA‐left (*p* = .003) between the two groups. The mean standard deviation of RD duration was 23.12 ± 11.20 days. Details are presented in Table 1.

**TABLE 1:** Conditions of participants included in the study

Condition	RD	NCs	*t*	*p*‐value*
Male/female	18/14	18/14	*N*/A	> .99
Age (years)	52.19 ± 6.97	51.17 ± 6.77	0.197	.892
Weight (kg)	66.42 ± 4.19	64.21 ± 5.11	0.214	.877
Handedness	32R	32R	*N*/A	>0.99
Duration of RD (days)	23.12 ± 11.20	*N*/A	*N*/A	*N*/A
Best‐corrected VA‐left eye	0.61 ± 0.21	1.02 ± 0.26	−3.322	.003
Best‐corrected VA‐right eye	0.63 ± 0.24	1.15 ± 0.29	−3.817	.001

Abbreviations:*N*/A, not applicable; NCs, normal controls; RD, retinal detachment; VA, visual acuity.

* *p* < .05, independent *t* tests comparing two groups, Data shown as mean ± standard deviation.

### 3.2 FCD analysis

**FIGURE 2:**
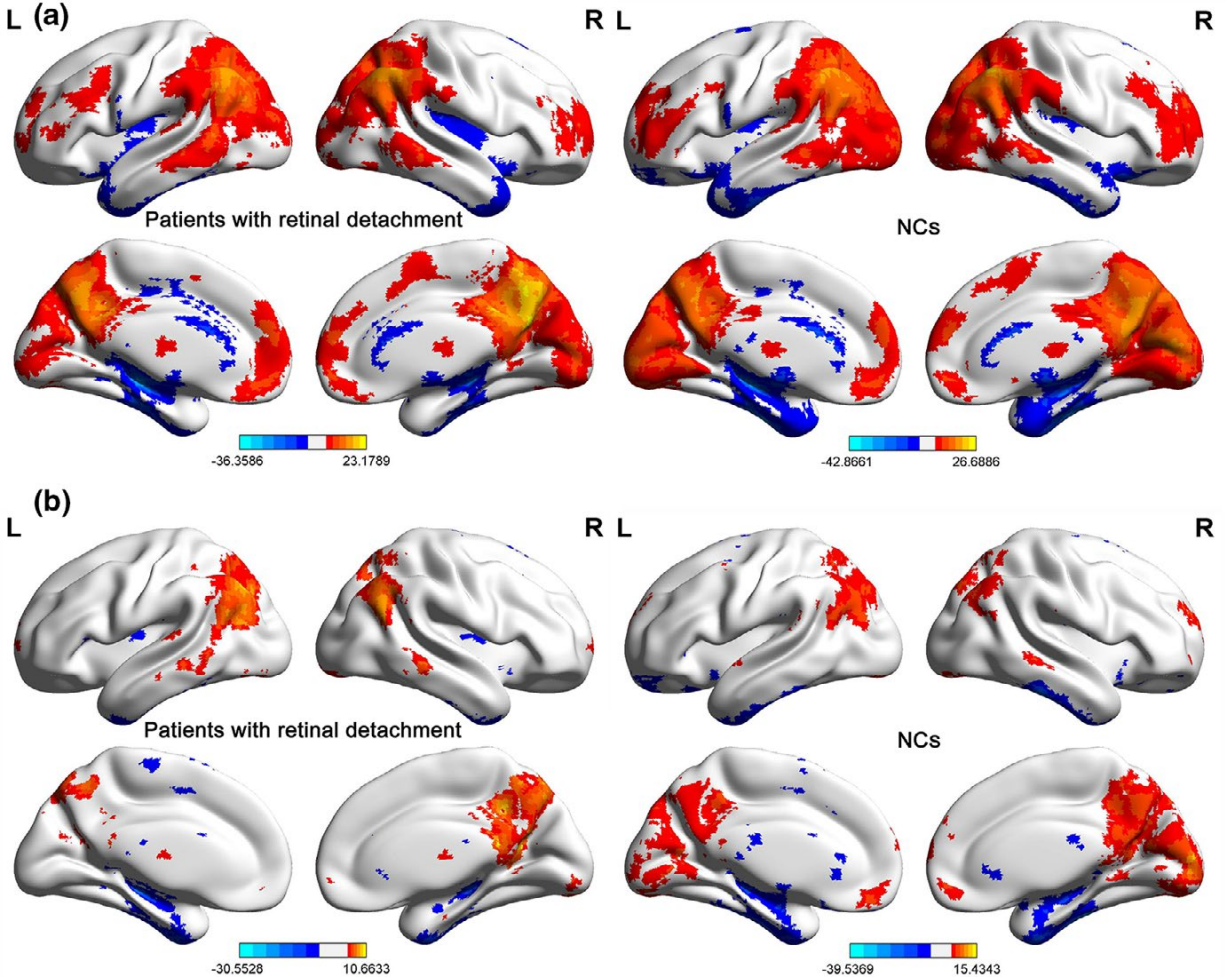
Spatial distribution of binarized lFCD and longFCD in patients with RD and NCs. Notes: Spatial distribution of binarized lFCD (a) and longFCD (b) in patients with RD and NCs The warm color areas denote higher values, and the cool color areas denote lower values in two groups. Abbreviations: lFCD, local functional connectivity density; longFCD, long‐range functional connectivity density; RD, retinal detachment; NCs, normal controls; L, left; R, right

**FIGURE 3:**
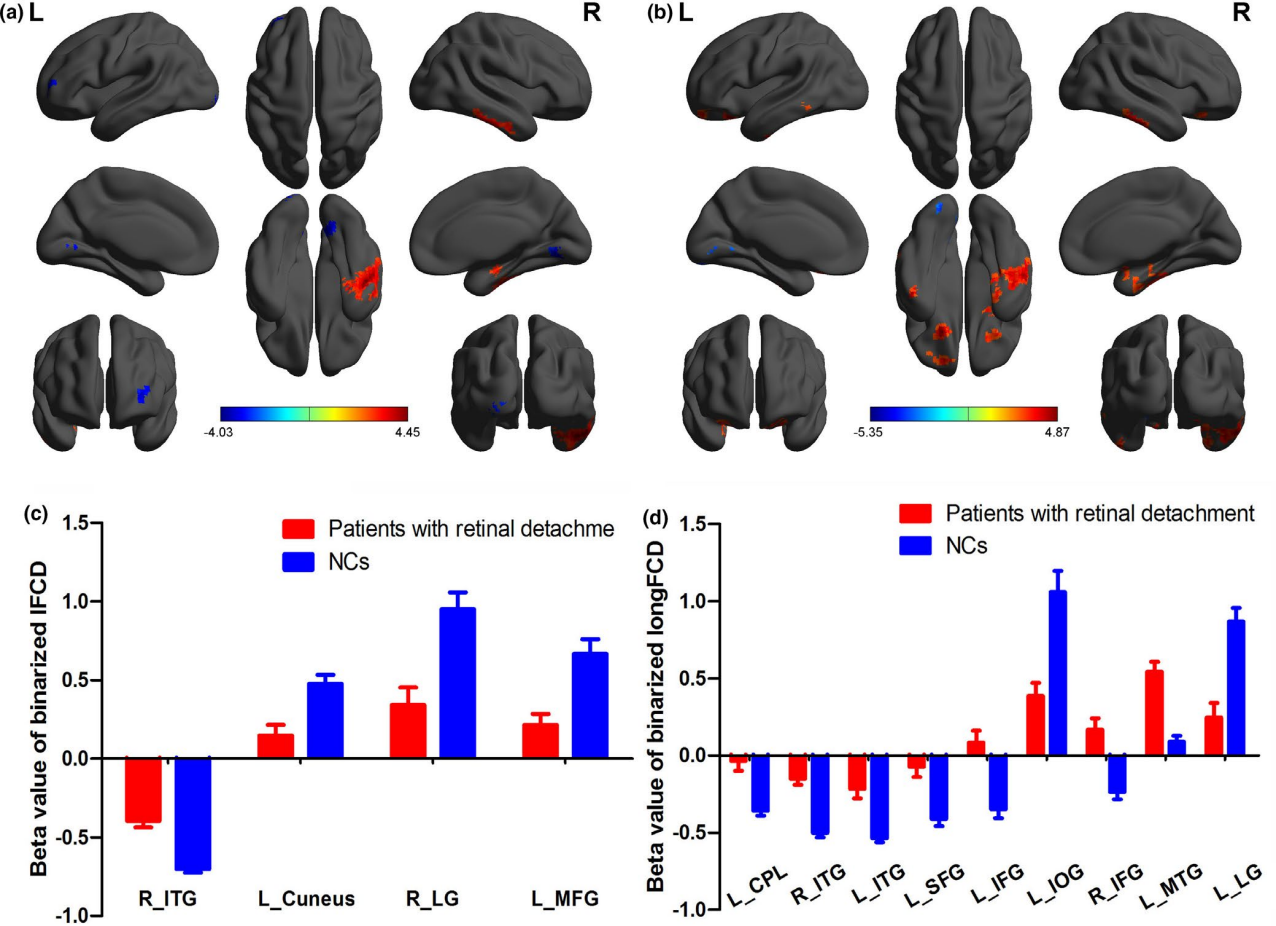
Binarized lFCD and longFCD differences between two groups. Notes: (a)/(b) Binarized lFCD/longFCD differences between patients with RDs and NCs. The warm color areas denote higher values, and the cool color areas denote lower values in RD patients in comparison with controls. (c)/(d) Binarized lFCD/ longFCD value of between‐group differences in different brain areas. The red bar denotes the value of patients with RD, and the blue bar denotes the value of NCs. Abbreviations: lFCD, local functional connectivity density; longFCD, long‐range functional connectivity density; RD, retinal detachment; NC, normal control; L, left; R, right

Compared with the NC group, middle‐age RD patients demonstrated increased lFCD values in the right inferior temporal gyrus (ITG), but decreased lFCD values in the left cuneus, right lingual gyrus, and left middle frontal gyrus (MFG) (Table 2, Figure 2a, Figure 3a and c). There were increased longFCD values in the bilateral inferior frontal gyri (IFG), left superior frontal gyrus (SFG), and bilateral ITG, left cerebellum posterior lobe (CPL), but decreased longFCD values in the left lingual gyrus and left inferior occipital gyrus (IOG) (Table 3, Figure 2b, Figure 3b and d).

**TABLE 2:** The binarized lFCD differences between patients with retinal detachment and NCs

Brain regions of peak coordinates	R/L	BA	Voxel size	*t*‐score of peak voxel	MNI coordinates
					X, Y, Z
Inferior temporal gyrus	R	20	376	4.4487	48, –21, –30
Cuneus	L	18	64	−4.0319	−15, –105, –6
Lingual gyrus	R	18	130	−3.8358	15, –72, –3
Middle frontal gyrus	L	10	52	−3.4367	−33, 57, 6

Notes

Between‐group differences in binarized lFCD thresholded at *r* = 0.3. We used thresholds of two‐tailed voxel‐wise *p* < .01 and cluster‐level *p* < .05, corrected for multiple comparisons by AlphaSim to determine the significant group differences.

Abbreviations: BA, Brodmann area; L, left; lFCD, local functional connectivity density;MNI, Montreal Neurological Institute; *N*/A, not applicable; R, right.

**TABLE 3:** The binarized longFCD differences between patients with retinal detachment and NCs

Brain regions of peak coordinates	R/L	BA	Voxel size	*t*‐score of peak voxel	MNI coordinates
					X, Y, Z
Cerebellum posterior lobe	L	*N*/A	178	3.9636	−24, –60, –63
Inferior temporal gyrus	R	20	399	4.8682	60, –36, –21
Inferior temporal gyrus	L	20	171	4.5204	−45, –18, –39
Superior frontal gyrus	L	11	57	3.7905	−18, 51, –21
Inferior frontal gyrus	L	11	79	4.7977	−18, 24, –21
Inferior occipital gyrus	L	18	50	−3.863	−24, –87, –18
Inferior frontal gyrus	R	11	48	4.0674	33, 27, –21
Middle temporal gyrus	L	21, 37	95	4.5634	−69, –51, 3
Lingual gyrus	L	18	67	−5.3517	−3, –66, 0

Notes

Between‐group differences in binarized longFCD thresholded at *r* = 0.3. We used thresholds of two‐tailed voxel‐wise *p* < .01 and cluster‐level *p* < .05, corrected for multiple comparisons by AlphaSim to determine the significant group differences.

Abbreviations: BA, Brodmann area; L, left; longFCD, long‐range functional connectivity density; MNI, Montreal Neurological Institute; *N*/A, not applicable; R, right.

### 3.3 ROC curve analysis

**FIGURE 4:**
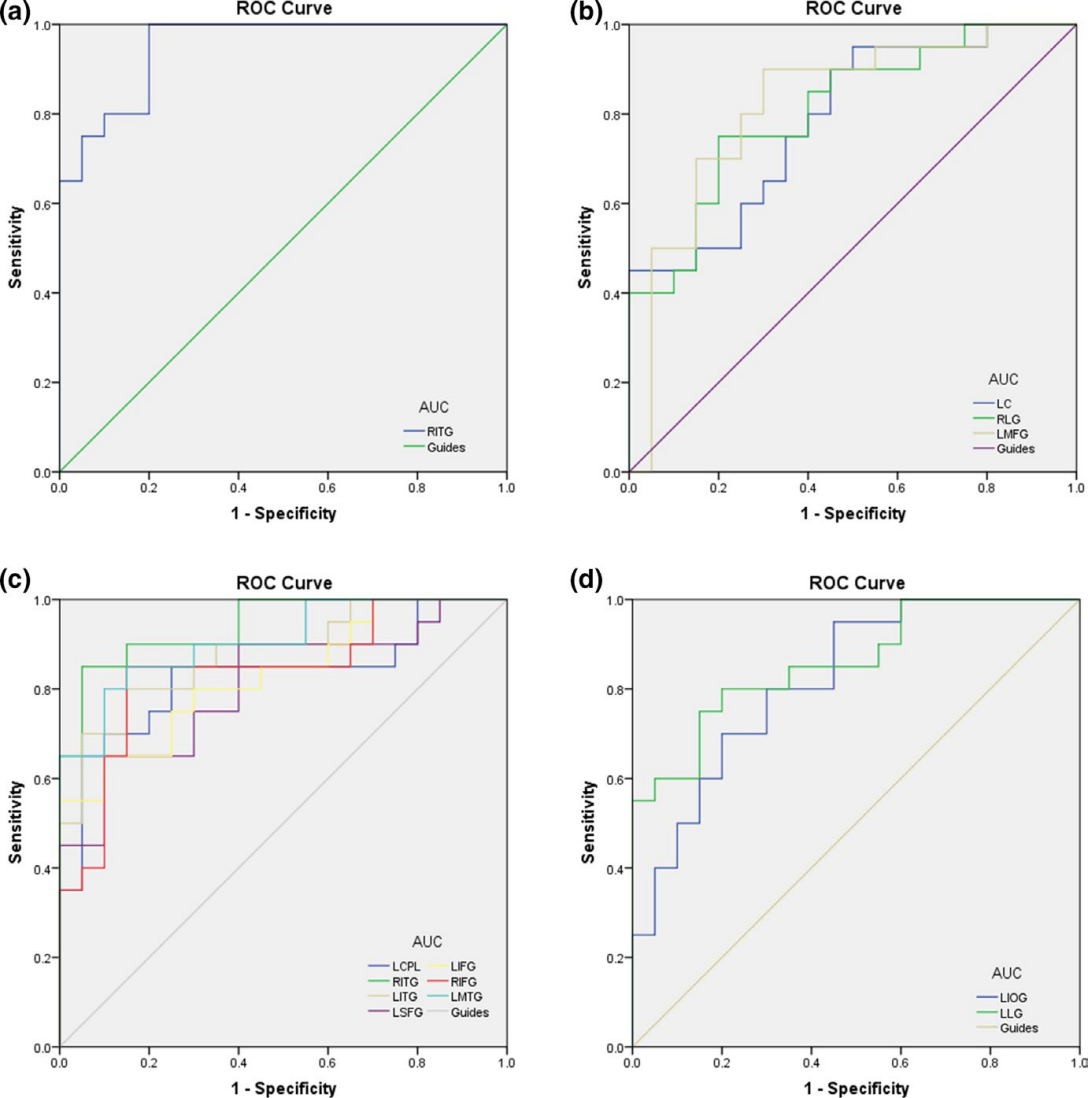
ROC curve analysis of the binarized lFCD and longFCD values for altered brain regions. Notes: (a) The area under the ROC curve of the binarized lFCD was 0.950 (*p* < .001, 95% CI: 0.890–1.000) for right inferior temporal gyrus. (b) The area under the ROC curve of the binarized lFCD was 0.788 (*p* = .002, 95% CI: 0.648–0.927) for left cuneus, 0.810 (*p* = .001, 95% CI: 0.678–0.942) for right lingual gyrus, and 0.823 (*p* < .001, 95% CI: 0.686–0.959) for left middle frontal gyrus. (c) The area under the ROC curve of the binarized longFCD was 0.827 (*p* < .001, 95% CI: 0.692–0.963) for left cerebellum posterior lobe, 0.943 (*p* < .001, 95% CI: 0.875–1.000) for right inferior temporal gyrus, 0.880 (*p* < .001, 95% CI: 0.775–0.985) for left inferior temporal gyrus, 0.807 (*p* < .001, 95% CI: 0.671–0.944) for left superior frontal gyrus, 0.830 (*p* < .001, 95% CI: 0.704–0.956) for left inferior frontal gyrus, 0.840 (*p* < .001, 95% CI: 0.712–0.968) for right inferior frontal gyrus, and 0.908 (*p* < .001, 95% CI: 0.817–0.998) for left middle temporal gyrus. (d) The area under the ROC curve of the binarized longFCD was 0.820 (*p* = .001, 95% CI: 0.692–0.948) for left inferior occipital gyrus and 0.860 (*p* < .001, 95% CI: 0.747–0.973) for left lingual gyrus. Abbreviations: lFCD, local functional connectivity density; longFCD, long‐range functional connectivity density; ROC, receiver operating characteristic

We hypothesized that the different lFCD and longFCD values could be useful diagnostic markers to differentiate between middle‐age RD group and NCs. ROC curve analysis was performed to test this assumption. The mean FCD values of distinct brain areas were collected and analyzed. The accuracy is low if the area under the curve (AUC) is 0.5–0.7, and when the AUC is 0.7–0.9, it means the accuracy is excellent. The individual AUCs of the binarized lFCD values for altered brain regions were as follows: right ITG (0.950, *p* < .001), left cuneus (0.788, *p* = .002), right lingual gyrus (0.810, *p* = .001), and left MFG (0.823, *p* < .001)(Figure 4a and b), and the individual AUCs of the binarized longFCD values were the left CPL (0.827, *p* < .00), right ITG (0.943, *p* < .001), left ITG (0.880, *p* < .001), left SFG (0.807, *p* < .001), left IFG (0.830, *p* < .001), right IFG (0.840, *p* < .001), left MTG (0.908, *p* < .001), left IOG (0.820, *p* = .001), and left lingual gyrus ( 0.860, *p* < .001) (Figure 4c and d).

## 4 DISCUSSION

**FIGURE 5:**
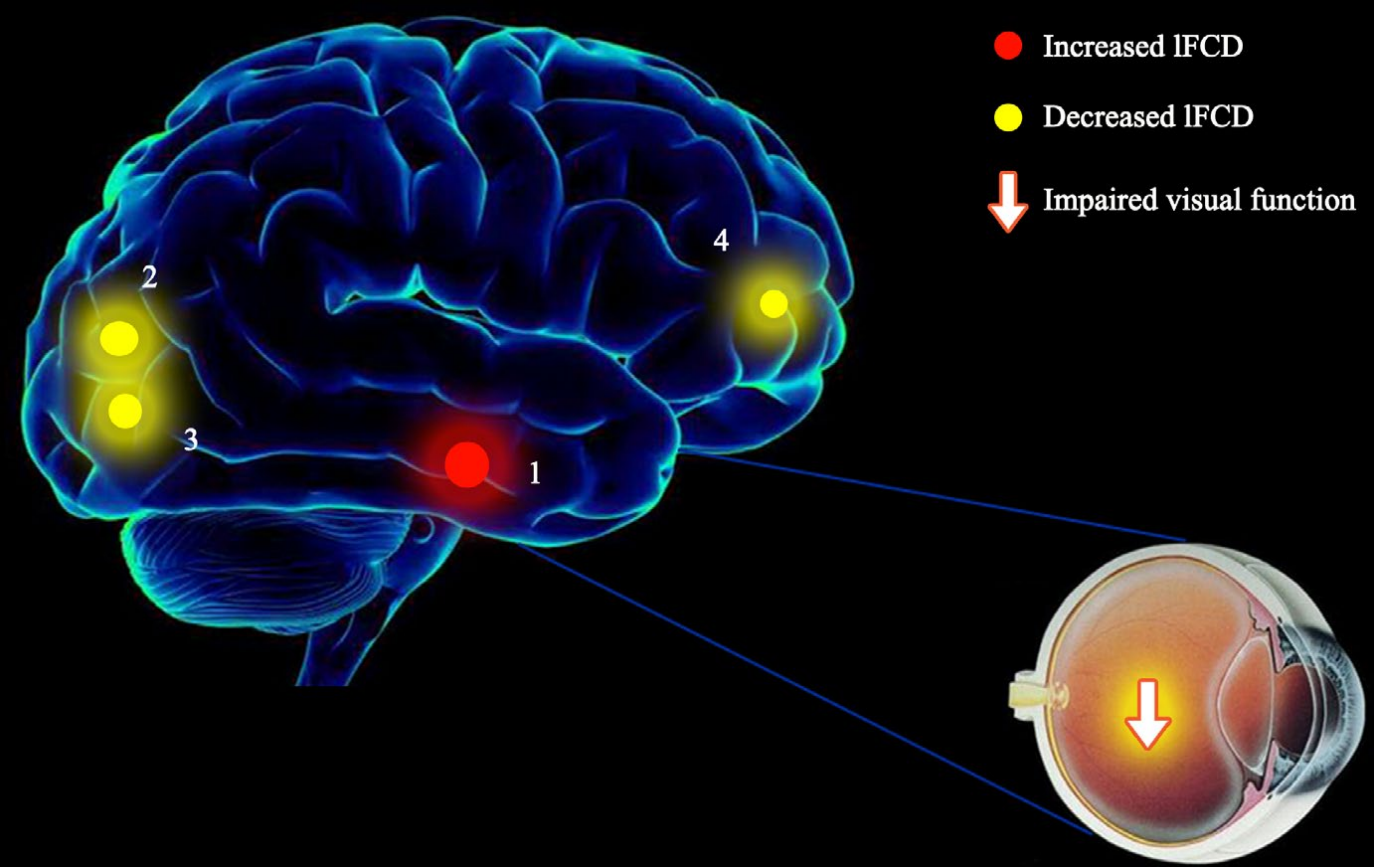
The binarized lFCD results of brain activity in the RD group. Notes: Compared with the NCs, the binarized lFCD of the following regions in the RD group was as follows: 1—right inferior temporal gyrus (*t* = 4.4487), 2—left cuneus (*t* = −4.0319), 3—right lingual gyrus (*t* = −3.8358), and 4—left middle frontal gyrus (*t* = −3.4367). Abbreviations: lFCD, local functional connectivity density; RD, retinal detachment; NCs, normal controls

**FIGURE 6:**
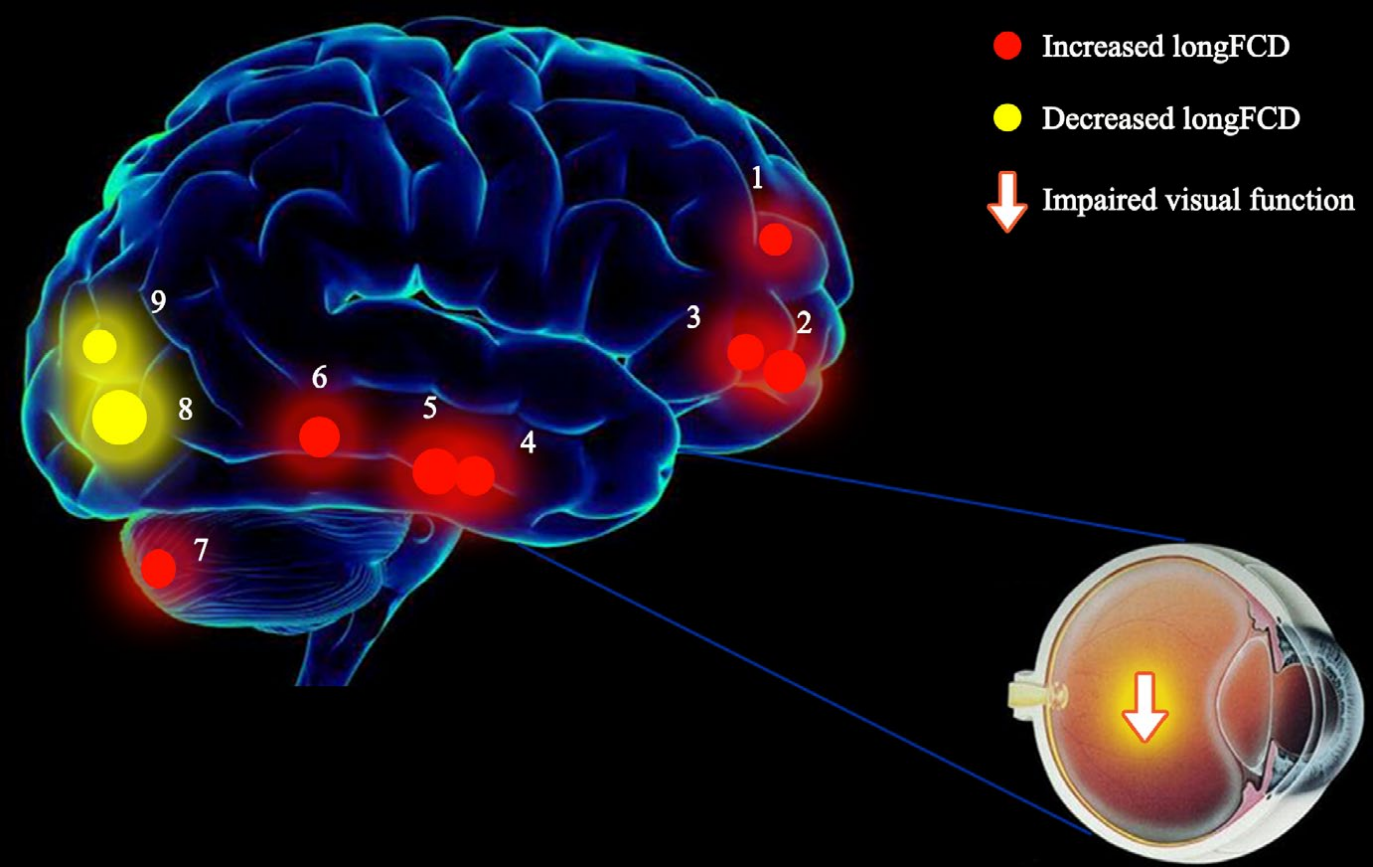
The binarized longFCD results of brain activity in the RD group. Notes: Compared with the NCs, the binarized longFCD of the following regions in the RD group was as follows: 1—left superior frontal gyrus (*t* = 3.7905), 2—left inferior frontal gyrus (*t* = −4.7977), 3—right inferior frontal gyrus (*t* = −4.0674), 4—left inferior temporal gyrus (*t* = −4.5204), 5—right inferior temporal gyrus (*t* = −4.8682), 6—left middle temporal gyrus (t = 4.5634), 7—left cerebellum posterior lobe (*t* = −3.9636), 8—left lingual gyrus (*t* = −5.3517), and 9—left inferior occipital gyrus (*t* = −3.8630). Abbreviations: longFCD, long‐range functional connectivity density; RD, retinal detachment; NCs, normal controls

Our results showed alterations in lFCD and longFCD in specific brain regions of patients with middle‐age RD (Figures 5 and 6). These changes may result in decreased VA, visual field defects, and poor resolution of external object size or color. The human visual system has highly efficient visual information processing. The pathway begins with light stimulation, which induces the nerve impulse to the visual formation. The visual information impulse is further transferred to the primary (V1) and secondary (V2) visual cortices, which consist of 19 and 18 zones, respectively. Previous studies of fMRI have demonstrated abnormal spontaneous activity in the RD patients. Decreased regional homogeneity values and abnormal rs‐FC in DMN subregions were observed in RD patients (Huang et al., 2017; Su et al., 2018), these reflected synchronous dysfunction of brain activity and might hindered the information connection of specific brain regions.

Compared with NCs, we found significant increases in FCD in some V2 areas (Brodmann area 18) including the left cuneus and right lingual gyrus for lFCD, and the left IOG and left lingual gyrus for longFCD. It is well known that the bilateral calcarine cortex in the occipital lobe receives input from the optic radiation, and the calcarine sulcus divides the interior of the posterior cerebral hemisphere into the cuneus and lingual gyrus. There is a strong theoretical and anatomical basis for decreased FCD in V2 due to RD. This result demonstrates that loss of visual information affects brain activity, which could help guide the development of RD treatments. It also helps explain the effect of losing stereoscopic vision in the corresponding functional areas of the brain. In addition to focusing on vision recovery, we should also observe and follow up brain function changes.

The loss of visual information in middle‐age patients with RD mainly affect vision; the visual field; and resolution of size, color, and spatial position. Previous studies reported beneficial behavioral effects of feature‐based attention (Ling, Liu, & Carrasco, 2009; Paltoglou et al., 2012) that have been attributed to modulation of neural activity in visual cortical areas specialized for processing the attended feature (Chawla, Rees, & Friston, 1999; Corbetta, Miezin, Dobmeyer, Shulman, & Petersen, 1991). However, basic features, such as color, are coded at multiple levels of the visual cortical processing hierarchy. In humans, color induces clear fMRI reactions in V1, V2, V3, V4, and ventral occipital (VO) lobes (Liu et al., 2005; D'Souza, Auer, Strasburger, Frahm, & Lee, 2011; Wade, Brewer, Rieger, & Wandell, 2002). Cone cells are damaged when RD affects the macula, and this decreases color perception. Bartsch (Bartsch et al., 2017) reported that attention to color is indexed by a sequence of modulations of the MRI brain response that are generated in the ventral extrastriate visual cortex. We also observe decreased FCD in these brain regions. To some extent, we believe these effects contribute to early disease symptoms.

Similarly, our brain processes numerosity, which is one of the basic visual attributes needed to acquire a detailed portrayal of the outside world (Dakin, Tibber, Greenwood, Kingdom, & Morgan, 2011; Durgin et al., 2008). Roggeman, Santens, Fias, and Verguts (2011) captured fMRI responses in the IOG and provided cogent proof for an occipitoparietal pathway conveying numerosity information. Another recent report (Fornaciai, Brannon, Woldorff, & Park, 2017) further demonstrated that numerosity processing begins at least by V2/V3. Compared with NCs, we observed lower longFCD in left IOG and other areas of V2 in RD patients, which may affect the effectiveness and accuracy of numerosity processing. Determining whether the effects of uni‐ or bilateral RD are the same will require further studies.

We also observed the decreased longFCD in the left CPL. This brain region has a vital function in elaborate motor coordination; for example, it can restrain involuntary movement though inhibitory neurotransmitters, especially GABA (Campbell, Meek, Zhang, & Bell, 2007). The CPL mainly receives input from the brainstem (reticular formation and inferior olivary nucleus) and cerebral cortex (Ramnani et al., 2012). Information received from sensorimotor associative cortices passes several parts of the brainstem and enters the retral section of the cerebellum through the medial peduncle (Kelly et al., 2003). Furthermore, there are anatomical relationship between the CPL and brain regions involved in higher cognitive functions. Middle‐age RD patients have difficulty performing fine movement because of decreased VA and visual field defect in the affected eye. The reduced longFCD values in the CPL of middle‐age RD patients may indicate reduced fine motor function.

Middle‐age patients with RD exhibited enhanced lFCD in the right ITG and higher longFCD in the bilateral ITG. The ITG is located below the MTG and is connected to the IOG, which is involved in ventral stream processing. This area is related to the portrayal of intricated object characteristics and the face perception (Haxby, Hoffman, & Gobbini, 2000). As the last location of the ventral cortical visual system, the ITG may be more active due to compensation for reduced VA in middle‐age RD patients.

The SFG makes up about a third of the frontal lobe. Based on fMRI experiments, Goldberg et al. proposed that the SFG was involved in self‐awareness and coordinated sensory system movements (Goldberg, Harel, & Malach, 2006). The SFG contributes to higher cognitive functions, especially working memory. Brodmann area 11 is involved in decision making (Rogers et al., 1999), processing rewards (Kringelbach et al., 2004), planning, encoding new information into long‐term memory (Frey et al., 2000), and reasoning. Recovery of VA after RD takes a long time, even with active treatment, and patients often experienced persistent loss of VA, especially for rhegmatogenous RD. Long‐term memory impairment may therefore stimulate the function of Brodmann area 11 in the SFG. This may explain the increased longFCD values in the left SFG and bilateral IFG (Brodmann area 11).

The MFG plays an important role in cognition and attention function (Chang et al., 2011). Decreased regional homogeneity values in the MFG were observed in RD patients, and this reflected local synchronization dysfunction in specific brain region (Huang et al., 2017). We found decreased lFCD in the left MFG. Compared with NCs, middle‐age RD patients lost different degrees of visual information. This could negatively impact cognition or attention, but the specific mechanism needs further study. We also observed enhanced longFCD in the bilateral IFG. Studies (Cha et al., 2016; Vanderhasselt, Kuhn, & De Raedt, 2013) suggest that the IFG may have a more complex role in emotion regulation; specifically, it is involved in evaluating stimulus meaning, which then informs the ventromedial prefrontal cortex to inhibit the amygdala. Due to the negative effects of visual information loss, RD patients may experience hypoemotivity or depression that increases FCD. The IFG is also considered a language region (Hobson et al., 2018). The left IFG has been identified as a neural crossroads between different types of information that are equally necessary for differentiating abstract and concepts. The right IFG plays a role in attentional switching (Hampshire et al., 2006) and appears to interact with areas of the temporal lobe implicated in memory (Aron et al., 2014). One group (Hampshire, Chamberlain, Monti, Duncan, & Owen, 2010) also reported that the right IFG is involved in inhibitory control and attentional control. We speculate that the adverse effects experienced by middle‐age patients with RD may influence their ability to deal with language and stimulate the brain to divert attention to overcome these effects, but studying this requires larger samples, more clinical data, and longer follow‐up.

## 5 LIMITATIONS

There are some limitations in this study. First of all, the emotion scales and cognitive assessments were not collected, previous studies have demonstrated abnormal spontaneous activity in patient with depression and cognitive impairment, and therefore, patients’ psychological and cognitive assessment data should be concerned and collected. Secondly, longitudinal studies are needed to investigate the influence of course of RD patients on lFCD and longFCD. In addition, we can assess the alterations of lFCD and longFCD in cured RD patients and compare them with that before any treatments, so as to observe whether the effect of FCD in RD patients is reversible. Finally, due to the small sample size of the study, the patients with RD were not further divided into groups according to right/left eye and area of RD, and we will include more behavioral performance data (e.g., electroretinogram and visual evoked potential) for analysis, larger samples should be included in future studies.

## 6 CONCLUSION

Middle‐age RD patients exhibited variations in binarized lFCD and longFCD in brain regions. Our results provide insight into the pathological mechanism of acute visual information loss in patients with RD.

## 7 CONFLICT OF INTEREST STATEMENT

This was not an industry‐supported study. The authors report no conflicts of interest in this work.

## 8 ETHICAL APPROVAL AND INFORMED CONSENT

All procedures performed in studies involving human participants were in accordance with the ethical standards of the First Affiliated Hospital of Nanchang University ethical committee and with the 1964 Helsinki declaration and its later amendments or comparable ethical standards.

## AUTHORS’ CONTRIBUTIONS

Shao Y and Lin Y designed and drafted the manuscript. Zhu P, Su T, and Zhou X analyzed the data. Li B, Shi W, Lin Q, Min Y, Yuan Q, and Ye L collected the data. Zhou Q revised the manuscript.

### PEER REVIEW

The peer review history for this article is available at https://publons.com/publon/10.1002/brb3.1783.

## Data Availability

The datasets generated during and/or analyzed during the current study are available from the corresponding author on reasonable request.
